# Ferrocene-1-carbaldehyde thio­semi­carbazone

**DOI:** 10.1107/S1600536810017605

**Published:** 2010-05-22

**Authors:** M. R. Vikneswaran, Siang Guan Teoh, Chin Sing Yeap, Hoong-Kun Fun

**Affiliations:** a School of Chemical Sciences, Universiti Sains Malaysia, 11800 USM, Penang, Malaysia; b X-ray Crystallography Unit, School of Physics, Universiti Sains Malaysia, 11800 USM, Penang, Malaysia

## Abstract

The asymmetric unit of the title compound, [Fe(C_5_H_5_)(C_7_H_8_N_3_S)], consists of two crystallographically independent mol­ecules, *A* and *B*. The cyclo­penta­dienyl (Cp) rings in both mol­ecules adopt an eclipsed conformation and are parallel to each other, forming dihedral angles of 2.5 (3) and 1.1 (3)°, respectively. The mean plane of the semicarbazone group is coplanar with the attached Cp ring in mol­ecule *A*, whereas it is twisted away in mol­ecule *B*. In the crystal structure, inter­molecular N—H⋯S hydrogen bonds link the mol­ecules into two-dimensional planes parallel to the *ab* plane. The structure is further consolidated by C—H⋯π inter­actions.

## Related literature

For related structures, see: Vikneswaran *et al.* (2009 ▶). For the synthesis of the title compound, see: Mariño *et al.* (2006 ▶). For the stability of the temperature controller used for the data collection, see: Cosier & Glazer (1986 ▶).
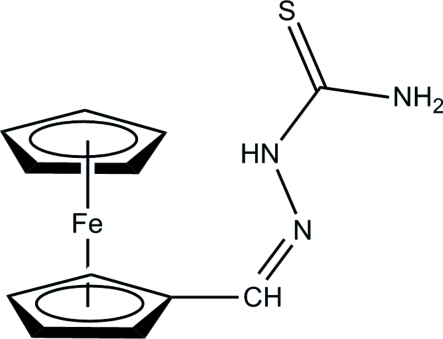


## Experimental

### 

#### Crystal data

[Fe(C_5_H_5_)(C_7_H_8_N_3_S)]
*M* *_r_* = 287.16
Triclinic, 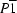

*a* = 5.8390 (1) Å
*b* = 12.7092 (3) Å
*c* = 16.7675 (4) Å
α = 94.447 (2)°
β = 97.965 (2)°
γ = 97.639 (2)°
*V* = 1215.51 (5) Å^3^
*Z* = 4
Mo *K*α radiation
μ = 1.39 mm^−1^
*T* = 100 K
0.30 × 0.11 × 0.05 mm

#### Data collection

Bruker SMART APEXII CCD area-detector diffractometer
Absorption correction: multi-scan (*SADABS*; Bruker, 2009 ▶) *T*_min_ = 0.681, *T*_max_ = 0.929
24408 measured reflections
5589 independent reflections
4024 reflections with *I* > 2σ(*I*)
*R*_int_ = 0.087

#### Refinement

*R*[*F* ^2^ > 2σ(*F* ^2^)] = 0.066
*wR*(*F* ^2^) = 0.169
*S* = 1.07
5589 reflections
307 parameters
H-atom parameters constrained
Δρ_max_ = 1.77 e Å^−3^
Δρ_min_ = −0.53 e Å^−3^

### 

Data collection: *APEX2* (Bruker, 2009 ▶); cell refinement: *SAINT* (Bruker, 2009 ▶); data reduction: *SAINT*; program(s) used to solve structure: *SHELXTL* (Sheldrick, 2008 ▶); program(s) used to refine structure: *SHELXTL*; molecular graphics: *SHELXTL*; software used to prepare material for publication: *SHELXTL* and *PLATON* (Spek, 2009 ▶).

## Supplementary Material

Crystal structure: contains datablocks global, I. DOI: 10.1107/S1600536810017605/rz2447sup1.cif

Structure factors: contains datablocks I. DOI: 10.1107/S1600536810017605/rz2447Isup2.hkl

Additional supplementary materials:  crystallographic information; 3D view; checkCIF report

## Figures and Tables

**Table 1:** Hydrogen-bond geometry (Å, °)

*D*—H⋯*A*	*D*—H	H⋯*A*	*D*⋯*A*	*D*—H⋯*A*
N2*A*—H2*AB*⋯S1*A*^i^	0.86	2.66	3.370 (4)	141
N3*A*—H3*AC*⋯S1*B*^ii^	0.86	2.46	3.295 (4)	165
N2*B*—H2*BB*⋯S1*A*^iii^	0.86	2.52	3.298 (4)	151
N3*B*—H3*BC*⋯S1*B*^iv^	0.86	2.47	3.323 (4)	173
C7*A*—H7*AA*⋯*Cg*1	0.98	2.90	3.668 (6)	136

Symmetry codes: (i) 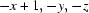; (ii) 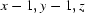; (iii) 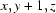; (iv) 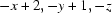.

**Fig. 1.**
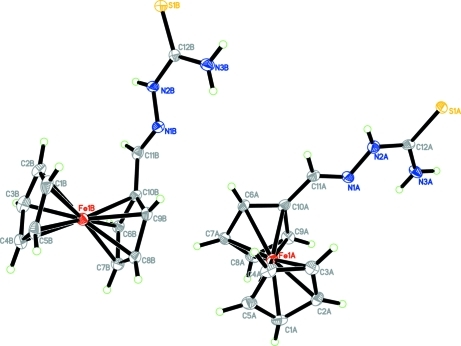
The molecular structure of the title compound, showing 50% probability displacement ellipsoids and the atom-numbering scheme.

**Fig. 2.**
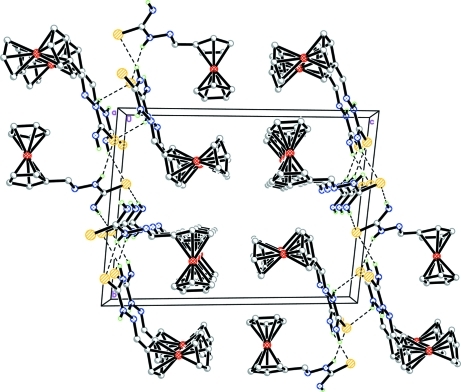
The crystal packing of the title compound, viewed along the a axis, showing 2-D layers parallel to ab plane. Hydrogen atoms not involved in hydrogen-bonding (dashed lines) are omitted for clarity.

## supplementary crystallographic information

### Comment

As a continuation of our research related to ferrocenyl thiosemicarbazones and their metal complexes, herein we report the crystal structure of formylferrocene thiosemicarbazone.

The asymmetric unit of title compound consists of two crystallographically independent molecules, *A* and *B* (Fig. 1). The geometric parameter are comparable to those observed in a closely related structure (Vikneswaran *et al.*, 2009). The Cp rings of each ferrocene residue are parallel, with dihedral angles of Cp1/Cp2 [C1A–C5A/C6A–C10A] = 2.5 (3)° and Cp3/Cp4 [C1B–C5B/C6B–C10B] = 1.1 (3)°. The Cp rings in both molecules adopt an eclipsed conformation [average torsion angles for C–Cg–Cg–C being 2.83 and 3.92°]. The mean plane of the semicarbazone group is coplanar with the attached Cp2 ring in molecule *A*, whereas the mean plane of semicarbazone group is twisted away from the attached Cp4 ring in molecule *B* with the dihedral angles between the mean plane and the Cp ring of 4.4 (2) and 33.3 (2)° respectively.

In the crystal structure, intermolecular N—H···S hydrogen bonds (Table 1) link the molecules into two-dimensional layers parallel to *ab* plane (Fig. 2). The crystal structure is further consolidated by C7A—H7AA···*Cg*1 interactions.

### Experimental

Formylferrocene thiosemicarbazone was prepared as described by Mariño *et al.* (2006). The single crystals were grown from an aqueous ethanol solution at room temperature in the dark.

### Refinement

All H-atoms were placed in calculated positions, with C–H = 0.93–0.98 Å and N–H = 0.86 Å and refined using a riding model, with *U*_iso_(H) = 1.2 *U*_eq_(C, N). The highest residual density peak is located 1.06 Å from atom Fe1B and the deepest hole is located 0.75 Å from atom Fe1A.

### Crystal data

**Table:**

[Fe(C_5_H_5_)(C_7_H_8_N_3_S)]	*Z* = 4
*M**_r_* = 287.16	*F*(000) = 592
Triclinic, *P*1	*D*_x_ = 1.569 Mg m^−^^3^
Hall symbol: -P 1	Mo *K*α radiation, λ = 0.71073 Å
*a* = 5.8390 (1) Å	Cell parameters from 3248 reflections
*b* = 12.7092 (3) Å	θ = 2.3–30.0°
*c* = 16.7675 (4) Å	µ = 1.39 mm^−^^1^
α = 94.447 (2)°	*T* = 100 K
β = 97.965 (2)°	Block, brown
γ = 97.639 (2)°	0.30 × 0.11 × 0.05 mm
*V* = 1215.51 (5) Å^3^	

### Data collection

**Table:**

Bruker SMART APEXII CCD area-detector diffractometer	5589 independent reflections
Radiation source: fine-focus sealed tube	4024 reflections with *I* > 2σ(*I*)
graphite	*R*_int_ = 0.087
φ and ω scans	θ_max_ = 27.5°, θ_min_ = 1.2°
Absorption correction: multi-scan (*SADABS*; Bruker, 2009)	*h* = −7→7
*T*_min_ = 0.681, *T*_max_ = 0.929	*k* = −16→16
24408 measured reflections	*l* = −21→21

### Refinement

**Table:**

Refinement on *F*^2^	Primary atom site location: structure-invariant direct methods
Least-squares matrix: full	Secondary atom site location: difference Fourier map
*R*[*F*^2^ > 2σ(*F*^2^)] = 0.066	Hydrogen site location: inferred from neighbouring sites
*wR*(*F*^2^) = 0.169	H-atom parameters constrained
*S* = 1.07	*w* = 1/[σ^2^(*F*_o_^2^) + (0.0849*P*)^2^ + 1.4801*P*] where *P* = (*F*_o_^2^ + 2*F*_c_^2^)/3
5589 reflections	(Δ/σ)_max_ < 0.001
307 parameters	Δρ_max_ = 1.77 e Å^−^^3^
0 restraints	Δρ_min_ = −0.53 e Å^−^^3^

### Special details

**Table:**

Experimental. The crystal was placed in the cold stream of an Oxford Cryosystems Cobra open-flow nitrogen cryostat (Cosier & Glazer, 1986) operating at 100.0 (1) K.
Geometry. All esds (except the esd in the dihedral angle between two l.s. planes) are estimated using the full covariance matrix. The cell esds are taken into account individually in the estimation of esds in distances, angles and torsion angles; correlations between esds in cell parameters are only used when they are defined by crystal symmetry. An approximate (isotropic) treatment of cell esds is used for estimating esds involving l.s. planes.
Refinement. Refinement of F^2^ against ALL reflections. The weighted R-factor wR and goodness of fit S are based on F^2^, conventional R-factors R are based on F, with F set to zero for negative F^2^. The threshold expression of F^2^ > 2sigma(F^2^) is used only for calculating R-factors(gt) etc. and is not relevant to the choice of reflections for refinement. R-factors based on F^2^ are statistically about twice as large as those based on F, and R- factors based on ALL data will be even larger.

### Fractional atomic coordinates and isotropic or equivalent isotropic displacement parameters (Å^2^)

**Table:**

	*x*	*y*	*z*	*U*_iso_*/*U*_eq_	
Fe1A	0.01430 (12)	0.26161 (5)	0.31434 (4)	0.01472 (18)	
S1A	0.3946 (2)	−0.15030 (10)	0.01954 (8)	0.0197 (3)	
N1A	0.0969 (7)	0.0658 (3)	0.1302 (2)	0.0172 (9)	
N2A	0.2533 (7)	0.0155 (3)	0.0929 (2)	0.0184 (9)	
H2AB	0.3870	0.0500	0.0882	0.022*	
N3A	−0.0087 (7)	−0.1375 (3)	0.0736 (2)	0.0198 (9)	
H3AB	−0.1016	−0.1040	0.0980	0.024*	
H3AC	−0.0498	−0.2036	0.0557	0.024*	
C1A	−0.0588 (9)	0.2347 (4)	0.4275 (3)	0.0212 (11)	
H1AA	−0.2125	0.2306	0.4452	0.025*	
C2A	0.0402 (9)	0.1482 (4)	0.3940 (3)	0.0194 (10)	
H2AA	−0.0351	0.0738	0.3839	0.023*	
C3A	0.2645 (9)	0.1878 (4)	0.3766 (3)	0.0202 (11)	
H3AA	0.3716	0.1458	0.3528	0.024*	
C4A	0.3071 (9)	0.3000 (4)	0.4004 (3)	0.0199 (11)	
H4AA	0.4483	0.3488	0.3957	0.024*	
C5A	0.1068 (10)	0.3281 (4)	0.4308 (3)	0.0235 (11)	
H5AA	0.0859	0.4001	0.4509	0.028*	
C6A	0.0681 (9)	0.3422 (4)	0.2154 (3)	0.0187 (10)	
H6AA	0.2115	0.3881	0.2087	0.022*	
C7A	−0.1234 (9)	0.3749 (4)	0.2508 (3)	0.0198 (11)	
H7AA	−0.1348	0.4476	0.2725	0.024*	
C8A	−0.2945 (9)	0.2840 (4)	0.2488 (3)	0.0204 (11)	
H8AA	−0.4440	0.2831	0.2692	0.024*	
C9A	−0.2107 (9)	0.1940 (4)	0.2135 (3)	0.0204 (11)	
H9AA	−0.2924	0.1206	0.2053	0.024*	
C10A	0.0119 (9)	0.2293 (4)	0.1916 (3)	0.0176 (10)	
C11A	0.1600 (9)	0.1663 (4)	0.1515 (3)	0.0176 (10)	
H11A	0.3034	0.1990	0.1409	0.021*	
C12A	0.1969 (8)	−0.0878 (4)	0.0638 (3)	0.0164 (10)	
Fe1B	0.31852 (12)	0.76679 (5)	0.33898 (4)	0.01544 (19)	
S1B	0.8033 (2)	0.62803 (10)	−0.02897 (7)	0.0184 (3)	
N1B	0.4716 (7)	0.6003 (3)	0.1573 (2)	0.0165 (8)	
N2B	0.5307 (7)	0.6233 (3)	0.0826 (2)	0.0171 (9)	
H2BB	0.4554	0.6651	0.0547	0.021*	
N3B	0.7899 (7)	0.5037 (3)	0.0921 (2)	0.0197 (9)	
H3BB	0.7344	0.4827	0.1340	0.024*	
H3BC	0.9013	0.4747	0.0750	0.024*	
C1B	0.6319 (9)	0.8646 (4)	0.3469 (3)	0.0240 (11)	
H1BA	0.7836	0.8411	0.3432	0.029*	
C2B	0.4734 (9)	0.8886 (4)	0.2811 (3)	0.0239 (11)	
H2BA	0.4962	0.8843	0.2242	0.029*	
C3B	0.2759 (10)	0.9180 (4)	0.3118 (3)	0.0249 (12)	
H3BA	0.1379	0.9386	0.2800	0.030*	
C4B	0.3125 (10)	0.9134 (4)	0.3974 (3)	0.0238 (11)	
H4BA	0.2049	0.9306	0.4348	0.029*	
C5B	0.5345 (9)	0.8807 (4)	0.4187 (3)	0.0230 (11)	
H5BA	0.6068	0.8708	0.4733	0.028*	
C6B	0.0264 (8)	0.6671 (4)	0.2828 (3)	0.0160 (10)	
H6BA	−0.1064	0.6878	0.2482	0.019*	
C7B	0.0491 (9)	0.6626 (4)	0.3678 (3)	0.0196 (10)	
H7BA	−0.0651	0.6808	0.4023	0.024*	
C8B	0.2653 (9)	0.6287 (4)	0.3946 (3)	0.0180 (10)	
H8BA	0.3261	0.6197	0.4507	0.022*	
C9B	0.3813 (9)	0.6118 (4)	0.3270 (3)	0.0160 (10)	
H9BA	0.5345	0.5880	0.3277	0.019*	
C10B	0.2328 (8)	0.6359 (4)	0.2570 (3)	0.0165 (10)	
C11B	0.2939 (9)	0.6408 (4)	0.1760 (3)	0.0168 (10)	
H11B	0.2040	0.6733	0.1376	0.020*	
C12B	0.7035 (8)	0.5809 (4)	0.0537 (3)	0.0145 (9)	

### Atomic displacement parameters (Å^2^)

**Table:**

	*U*^11^	*U*^22^	*U*^33^	*U*^12^	*U*^13^	*U*^23^
Fe1A	0.0161 (4)	0.0153 (4)	0.0130 (3)	0.0028 (3)	0.0020 (3)	0.0026 (3)
S1A	0.0174 (6)	0.0182 (6)	0.0271 (6)	0.0065 (5)	0.0101 (5)	0.0059 (5)
N1A	0.015 (2)	0.019 (2)	0.019 (2)	0.0050 (17)	0.0053 (17)	0.0022 (16)
N2A	0.016 (2)	0.018 (2)	0.022 (2)	0.0024 (17)	0.0038 (17)	0.0039 (17)
N3A	0.014 (2)	0.020 (2)	0.024 (2)	0.0008 (18)	0.0059 (17)	−0.0045 (17)
C1A	0.022 (3)	0.030 (3)	0.013 (2)	0.005 (2)	0.005 (2)	0.006 (2)
C2A	0.022 (3)	0.019 (2)	0.018 (2)	0.004 (2)	0.000 (2)	0.0065 (19)
C3A	0.019 (3)	0.024 (3)	0.018 (2)	0.006 (2)	0.000 (2)	0.003 (2)
C4A	0.015 (2)	0.025 (3)	0.017 (2)	−0.003 (2)	−0.0027 (19)	0.003 (2)
C5A	0.032 (3)	0.027 (3)	0.013 (2)	0.009 (2)	0.003 (2)	0.003 (2)
C6A	0.025 (3)	0.017 (2)	0.015 (2)	0.005 (2)	0.003 (2)	0.0057 (18)
C7A	0.026 (3)	0.020 (2)	0.016 (2)	0.011 (2)	0.004 (2)	0.0047 (19)
C8A	0.019 (3)	0.027 (3)	0.015 (2)	0.007 (2)	0.002 (2)	0.000 (2)
C9A	0.018 (3)	0.023 (3)	0.019 (2)	0.004 (2)	0.000 (2)	0.000 (2)
C10A	0.020 (3)	0.017 (2)	0.014 (2)	0.000 (2)	0.0013 (19)	0.0034 (18)
C11A	0.016 (2)	0.022 (3)	0.016 (2)	0.004 (2)	0.0033 (19)	0.0053 (19)
C12A	0.013 (2)	0.020 (2)	0.017 (2)	0.000 (2)	0.0042 (19)	0.0040 (19)
Fe1B	0.0157 (4)	0.0154 (4)	0.0150 (3)	0.0021 (3)	0.0022 (3)	0.0008 (3)
S1B	0.0210 (6)	0.0173 (6)	0.0186 (6)	0.0033 (5)	0.0078 (5)	0.0030 (5)
N1B	0.018 (2)	0.015 (2)	0.0164 (19)	0.0006 (17)	0.0045 (17)	0.0016 (15)
N2B	0.017 (2)	0.017 (2)	0.020 (2)	0.0065 (17)	0.0082 (17)	0.0051 (16)
N3B	0.019 (2)	0.022 (2)	0.021 (2)	0.0073 (18)	0.0076 (17)	0.0045 (17)
C1B	0.019 (3)	0.014 (2)	0.037 (3)	−0.002 (2)	0.002 (2)	0.001 (2)
C2B	0.026 (3)	0.019 (3)	0.027 (3)	−0.002 (2)	0.009 (2)	0.006 (2)
C3B	0.025 (3)	0.020 (3)	0.029 (3)	0.001 (2)	−0.001 (2)	0.006 (2)
C4B	0.028 (3)	0.017 (2)	0.027 (3)	0.003 (2)	0.009 (2)	−0.001 (2)
C5B	0.027 (3)	0.014 (2)	0.024 (3)	−0.001 (2)	−0.004 (2)	−0.001 (2)
C6B	0.013 (2)	0.015 (2)	0.020 (2)	0.0012 (19)	0.0028 (19)	0.0029 (18)
C7B	0.018 (3)	0.018 (2)	0.021 (2)	−0.005 (2)	0.005 (2)	−0.0033 (19)
C8B	0.019 (2)	0.015 (2)	0.021 (2)	0.001 (2)	0.005 (2)	0.0024 (19)
C9B	0.017 (2)	0.013 (2)	0.018 (2)	0.0013 (19)	0.0021 (19)	0.0001 (18)
C10B	0.017 (2)	0.015 (2)	0.018 (2)	−0.0007 (19)	0.0049 (19)	0.0030 (18)
C11B	0.018 (2)	0.014 (2)	0.017 (2)	−0.002 (2)	−0.0010 (19)	0.0013 (18)
C12B	0.014 (2)	0.014 (2)	0.014 (2)	0.0012 (19)	0.0000 (18)	−0.0002 (17)

### Geometric parameters (Å, °)

**Table:**

Fe1A—C5A	2.036 (5)	Fe1B—C10B	2.034 (5)
Fe1A—C7A	2.040 (5)	Fe1B—C4B	2.043 (5)
Fe1A—C8A	2.043 (5)	Fe1B—C6B	2.048 (5)
Fe1A—C9A	2.045 (5)	Fe1B—C3B	2.049 (5)
Fe1A—C2A	2.045 (5)	Fe1B—C9B	2.051 (5)
Fe1A—C1A	2.045 (5)	Fe1B—C1B	2.053 (5)
Fe1A—C3A	2.049 (5)	Fe1B—C7B	2.054 (5)
Fe1A—C6A	2.054 (5)	Fe1B—C5B	2.054 (5)
Fe1A—C4A	2.057 (5)	Fe1B—C8B	2.058 (5)
Fe1A—C10A	2.065 (5)	Fe1B—C2B	2.064 (5)
S1A—C12A	1.704 (5)	S1B—C12B	1.697 (5)
N1A—C11A	1.289 (6)	N1B—C11B	1.283 (6)
N1A—N2A	1.373 (5)	N1B—N2B	1.386 (5)
N2A—C12A	1.346 (6)	N2B—C12B	1.336 (6)
N2A—H2AB	0.8600	N2B—H2BB	0.8600
N3A—C12A	1.318 (6)	N3B—C12B	1.331 (6)
N3A—H3AB	0.8600	N3B—H3BB	0.8600
N3A—H3AC	0.8600	N3B—H3BC	0.8600
C1A—C5A	1.419 (8)	C1B—C5B	1.413 (8)
C1A—C2A	1.422 (7)	C1B—C2B	1.417 (7)
C1A—H1AA	0.9800	C1B—H1BA	0.9800
C2A—C3A	1.416 (7)	C2B—C3B	1.408 (8)
C2A—H2AA	0.9800	C2B—H2BA	0.9800
C3A—C4A	1.430 (7)	C3B—C4B	1.429 (7)
C3A—H3AA	0.9800	C3B—H3BA	0.9800
C4A—C5A	1.415 (7)	C4B—C5B	1.420 (7)
C4A—H4AA	0.9800	C4B—H4BA	0.9800
C5A—H5AA	0.9800	C5B—H5BA	0.9800
C6A—C7A	1.430 (7)	C6B—C7B	1.420 (7)
C6A—C10A	1.440 (7)	C6B—C10B	1.429 (7)
C6A—H6AA	0.9800	C6B—H6BA	0.9800
C7A—C8A	1.418 (7)	C7B—C8B	1.413 (7)
C7A—H7AA	0.9800	C7B—H7BA	0.9800
C8A—C9A	1.423 (7)	C8B—C9B	1.416 (7)
C8A—H8AA	0.9800	C8B—H8BA	0.9800
C9A—C10A	1.424 (7)	C9B—C10B	1.437 (6)
C9A—H9AA	0.9800	C9B—H9BA	0.9800
C10A—C11A	1.450 (7)	C10B—C11B	1.456 (7)
C11A—H11A	0.9300	C11B—H11B	0.9300
			
C5A—Fe1A—C7A	106.2 (2)	C10B—Fe1B—C4B	160.8 (2)
C5A—Fe1A—C8A	120.3 (2)	C10B—Fe1B—C6B	40.97 (19)
C7A—Fe1A—C8A	40.6 (2)	C4B—Fe1B—C6B	124.1 (2)
C5A—Fe1A—C9A	156.0 (2)	C10B—Fe1B—C3B	123.6 (2)
C7A—Fe1A—C9A	68.7 (2)	C4B—Fe1B—C3B	40.9 (2)
C8A—Fe1A—C9A	40.72 (19)	C6B—Fe1B—C3B	107.1 (2)
C5A—Fe1A—C2A	68.2 (2)	C10B—Fe1B—C9B	41.19 (18)
C7A—Fe1A—C2A	159.1 (2)	C4B—Fe1B—C9B	156.8 (2)
C8A—Fe1A—C2A	123.3 (2)	C6B—Fe1B—C9B	68.92 (19)
C9A—Fe1A—C2A	107.7 (2)	C3B—Fe1B—C9B	160.8 (2)
C5A—Fe1A—C1A	40.7 (2)	C10B—Fe1B—C1B	121.3 (2)
C7A—Fe1A—C1A	122.1 (2)	C4B—Fe1B—C1B	68.0 (2)
C8A—Fe1A—C1A	105.8 (2)	C6B—Fe1B—C1B	156.5 (2)
C9A—Fe1A—C1A	120.7 (2)	C3B—Fe1B—C1B	67.7 (2)
C2A—Fe1A—C1A	40.7 (2)	C9B—Fe1B—C1B	108.0 (2)
C5A—Fe1A—C3A	68.4 (2)	C10B—Fe1B—C7B	68.31 (19)
C7A—Fe1A—C3A	158.1 (2)	C4B—Fe1B—C7B	108.2 (2)
C8A—Fe1A—C3A	160.6 (2)	C6B—Fe1B—C7B	40.49 (18)
C9A—Fe1A—C3A	124.9 (2)	C3B—Fe1B—C7B	121.8 (2)
C2A—Fe1A—C3A	40.5 (2)	C9B—Fe1B—C7B	68.1 (2)
C1A—Fe1A—C3A	68.5 (2)	C1B—Fe1B—C7B	161.8 (2)
C5A—Fe1A—C6A	123.7 (2)	C10B—Fe1B—C5B	156.8 (2)
C7A—Fe1A—C6A	40.89 (19)	C4B—Fe1B—C5B	40.6 (2)
C8A—Fe1A—C6A	68.5 (2)	C6B—Fe1B—C5B	161.3 (2)
C9A—Fe1A—C6A	68.8 (2)	C3B—Fe1B—C5B	68.2 (2)
C2A—Fe1A—C6A	158.8 (2)	C9B—Fe1B—C5B	121.6 (2)
C1A—Fe1A—C6A	159.4 (2)	C1B—Fe1B—C5B	40.3 (2)
C3A—Fe1A—C6A	123.2 (2)	C7B—Fe1B—C5B	125.3 (2)
C5A—Fe1A—C4A	40.5 (2)	C10B—Fe1B—C8B	68.31 (19)
C7A—Fe1A—C4A	121.5 (2)	C4B—Fe1B—C8B	122.1 (2)
C8A—Fe1A—C4A	156.3 (2)	C6B—Fe1B—C8B	68.15 (19)
C9A—Fe1A—C4A	162.1 (2)	C3B—Fe1B—C8B	157.3 (2)
C2A—Fe1A—C4A	68.2 (2)	C9B—Fe1B—C8B	40.31 (19)
C1A—Fe1A—C4A	68.4 (2)	C1B—Fe1B—C8B	125.5 (2)
C3A—Fe1A—C4A	40.8 (2)	C7B—Fe1B—C8B	40.21 (19)
C6A—Fe1A—C4A	108.4 (2)	C5B—Fe1B—C8B	108.8 (2)
C5A—Fe1A—C10A	161.4 (2)	C10B—Fe1B—C2B	107.3 (2)
C7A—Fe1A—C10A	68.63 (19)	C4B—Fe1B—C2B	68.0 (2)
C8A—Fe1A—C10A	68.21 (19)	C6B—Fe1B—C2B	121.0 (2)
C9A—Fe1A—C10A	40.5 (2)	C3B—Fe1B—C2B	40.0 (2)
C2A—Fe1A—C10A	122.9 (2)	C9B—Fe1B—C2B	124.7 (2)
C1A—Fe1A—C10A	157.2 (2)	C1B—Fe1B—C2B	40.3 (2)
C3A—Fe1A—C10A	109.4 (2)	C7B—Fe1B—C2B	156.6 (2)
C6A—Fe1A—C10A	40.94 (18)	C5B—Fe1B—C2B	67.8 (2)
C4A—Fe1A—C10A	125.8 (2)	C8B—Fe1B—C2B	161.6 (2)
C11A—N1A—N2A	115.4 (4)	C11B—N1B—N2B	114.4 (4)
C12A—N2A—N1A	119.9 (4)	C12B—N2B—N1B	120.5 (4)
C12A—N2A—H2AB	120.0	C12B—N2B—H2BB	119.7
N1A—N2A—H2AB	120.0	N1B—N2B—H2BB	119.7
C12A—N3A—H3AB	120.0	C12B—N3B—H3BB	120.0
C12A—N3A—H3AC	120.0	C12B—N3B—H3BC	120.0
H3AB—N3A—H3AC	120.0	H3BB—N3B—H3BC	120.0
C5A—C1A—C2A	107.3 (5)	C5B—C1B—C2B	108.4 (5)
C5A—C1A—Fe1A	69.3 (3)	C5B—C1B—Fe1B	69.9 (3)
C2A—C1A—Fe1A	69.7 (3)	C2B—C1B—Fe1B	70.3 (3)
C5A—C1A—H1AA	126.4	C5B—C1B—H1BA	125.8
C2A—C1A—H1AA	126.4	C2B—C1B—H1BA	125.8
Fe1A—C1A—H1AA	126.4	Fe1B—C1B—H1BA	125.8
C3A—C2A—C1A	108.6 (5)	C3B—C2B—C1B	108.0 (5)
C3A—C2A—Fe1A	69.9 (3)	C3B—C2B—Fe1B	69.4 (3)
C1A—C2A—Fe1A	69.7 (3)	C1B—C2B—Fe1B	69.4 (3)
C3A—C2A—H2AA	125.7	C3B—C2B—H2BA	126.0
C1A—C2A—H2AA	125.7	C1B—C2B—H2BA	126.0
Fe1A—C2A—H2AA	125.7	Fe1B—C2B—H2BA	126.0
C2A—C3A—C4A	107.7 (4)	C2B—C3B—C4B	108.1 (5)
C2A—C3A—Fe1A	69.6 (3)	C2B—C3B—Fe1B	70.6 (3)
C4A—C3A—Fe1A	69.9 (3)	C4B—C3B—Fe1B	69.4 (3)
C2A—C3A—H3AA	126.1	C2B—C3B—H3BA	126.0
C4A—C3A—H3AA	126.1	C4B—C3B—H3BA	126.0
Fe1A—C3A—H3AA	126.1	Fe1B—C3B—H3BA	126.0
C5A—C4A—C3A	107.6 (5)	C5B—C4B—C3B	107.6 (5)
C5A—C4A—Fe1A	69.0 (3)	C5B—C4B—Fe1B	70.1 (3)
C3A—C4A—Fe1A	69.3 (3)	C3B—C4B—Fe1B	69.8 (3)
C5A—C4A—H4AA	126.2	C5B—C4B—H4BA	126.2
C3A—C4A—H4AA	126.2	C3B—C4B—H4BA	126.2
Fe1A—C4A—H4AA	126.2	Fe1B—C4B—H4BA	126.2
C4A—C5A—C1A	108.8 (5)	C1B—C5B—C4B	107.8 (5)
C4A—C5A—Fe1A	70.5 (3)	C1B—C5B—Fe1B	69.8 (3)
C1A—C5A—Fe1A	70.0 (3)	C4B—C5B—Fe1B	69.3 (3)
C4A—C5A—H5AA	125.6	C1B—C5B—H5BA	126.1
C1A—C5A—H5AA	125.6	C4B—C5B—H5BA	126.1
Fe1A—C5A—H5AA	125.6	Fe1B—C5B—H5BA	126.1
C7A—C6A—C10A	107.5 (5)	C7B—C6B—C10B	107.4 (4)
C7A—C6A—Fe1A	69.0 (3)	C7B—C6B—Fe1B	70.0 (3)
C10A—C6A—Fe1A	69.9 (3)	C10B—C6B—Fe1B	69.0 (3)
C7A—C6A—H6AA	126.3	C7B—C6B—H6BA	126.3
C10A—C6A—H6AA	126.3	C10B—C6B—H6BA	126.3
Fe1A—C6A—H6AA	126.3	Fe1B—C6B—H6BA	126.3
C8A—C7A—C6A	108.1 (4)	C8B—C7B—C6B	108.6 (4)
C8A—C7A—Fe1A	69.8 (3)	C8B—C7B—Fe1B	70.1 (3)
C6A—C7A—Fe1A	70.1 (3)	C6B—C7B—Fe1B	69.5 (3)
C8A—C7A—H7AA	125.9	C8B—C7B—H7BA	125.7
C6A—C7A—H7AA	125.9	C6B—C7B—H7BA	125.7
Fe1A—C7A—H7AA	125.9	Fe1B—C7B—H7BA	125.7
C7A—C8A—C9A	108.5 (4)	C7B—C8B—C9B	108.7 (4)
C7A—C8A—Fe1A	69.6 (3)	C7B—C8B—Fe1B	69.7 (3)
C9A—C8A—Fe1A	69.7 (3)	C9B—C8B—Fe1B	69.6 (3)
C7A—C8A—H8AA	125.7	C7B—C8B—H8BA	125.7
C9A—C8A—H8AA	125.7	C9B—C8B—H8BA	125.7
Fe1A—C8A—H8AA	125.7	Fe1B—C8B—H8BA	125.7
C8A—C9A—C10A	108.1 (5)	C8B—C9B—C10B	107.3 (4)
C8A—C9A—Fe1A	69.6 (3)	C8B—C9B—Fe1B	70.1 (3)
C10A—C9A—Fe1A	70.5 (3)	C10B—C9B—Fe1B	68.8 (3)
C8A—C9A—H9AA	126.0	C8B—C9B—H9BA	126.4
C10A—C9A—H9AA	126.0	C10B—C9B—H9BA	126.4
Fe1A—C9A—H9AA	126.0	Fe1B—C9B—H9BA	126.4
C9A—C10A—C6A	107.8 (4)	C6B—C10B—C9B	108.1 (4)
C9A—C10A—C11A	127.7 (5)	C6B—C10B—C11B	125.6 (4)
C6A—C10A—C11A	124.4 (5)	C9B—C10B—C11B	125.9 (4)
C9A—C10A—Fe1A	69.0 (3)	C6B—C10B—Fe1B	70.0 (3)
C6A—C10A—Fe1A	69.1 (3)	C9B—C10B—Fe1B	70.0 (3)
C11A—C10A—Fe1A	128.0 (3)	C11B—C10B—Fe1B	119.8 (3)
N1A—C11A—C10A	121.3 (5)	N1B—C11B—C10B	120.1 (4)
N1A—C11A—H11A	119.4	N1B—C11B—H11B	119.9
C10A—C11A—H11A	119.4	C10B—C11B—H11B	119.9
N3A—C12A—N2A	117.7 (4)	N3B—C12B—N2B	117.7 (4)
N3A—C12A—S1A	122.7 (4)	N3B—C12B—S1B	123.3 (4)
N2A—C12A—S1A	119.6 (4)	N2B—C12B—S1B	118.9 (3)
			
C11A—N1A—N2A—C12A	−175.7 (4)	C11B—N1B—N2B—C12B	−176.0 (4)
C7A—Fe1A—C1A—C5A	−77.1 (4)	C10B—Fe1B—C1B—C5B	−161.2 (3)
C8A—Fe1A—C1A—C5A	−118.3 (3)	C4B—Fe1B—C1B—C5B	37.8 (3)
C9A—Fe1A—C1A—C5A	−159.9 (3)	C6B—Fe1B—C1B—C5B	163.7 (4)
C2A—Fe1A—C1A—C5A	118.6 (4)	C3B—Fe1B—C1B—C5B	82.1 (3)
C3A—Fe1A—C1A—C5A	81.4 (3)	C9B—Fe1B—C1B—C5B	−117.9 (3)
C6A—Fe1A—C1A—C5A	−47.4 (7)	C7B—Fe1B—C1B—C5B	−43.4 (8)
C4A—Fe1A—C1A—C5A	37.4 (3)	C8B—Fe1B—C1B—C5B	−76.7 (4)
C10A—Fe1A—C1A—C5A	170.4 (5)	C2B—Fe1B—C1B—C5B	119.3 (4)
C5A—Fe1A—C1A—C2A	−118.6 (4)	C10B—Fe1B—C1B—C2B	79.6 (3)
C7A—Fe1A—C1A—C2A	164.3 (3)	C4B—Fe1B—C1B—C2B	−81.5 (3)
C8A—Fe1A—C1A—C2A	123.1 (3)	C6B—Fe1B—C1B—C2B	44.4 (6)
C9A—Fe1A—C1A—C2A	81.5 (3)	C3B—Fe1B—C1B—C2B	−37.2 (3)
C3A—Fe1A—C1A—C2A	−37.2 (3)	C9B—Fe1B—C1B—C2B	122.8 (3)
C6A—Fe1A—C1A—C2A	−166.0 (5)	C7B—Fe1B—C1B—C2B	−162.6 (6)
C4A—Fe1A—C1A—C2A	−81.2 (3)	C5B—Fe1B—C1B—C2B	−119.3 (4)
C10A—Fe1A—C1A—C2A	51.8 (6)	C8B—Fe1B—C1B—C2B	164.0 (3)
C5A—C1A—C2A—C3A	−0.1 (5)	C5B—C1B—C2B—C3B	−0.9 (6)
Fe1A—C1A—C2A—C3A	59.3 (3)	Fe1B—C1B—C2B—C3B	58.9 (4)
C5A—C1A—C2A—Fe1A	−59.3 (3)	C5B—C1B—C2B—Fe1B	−59.7 (4)
C5A—Fe1A—C2A—C3A	−81.8 (3)	C10B—Fe1B—C2B—C3B	122.0 (3)
C7A—Fe1A—C2A—C3A	−160.0 (5)	C4B—Fe1B—C2B—C3B	−38.1 (3)
C8A—Fe1A—C2A—C3A	165.5 (3)	C6B—Fe1B—C2B—C3B	79.4 (4)
C9A—Fe1A—C2A—C3A	123.4 (3)	C9B—Fe1B—C2B—C3B	164.0 (3)
C1A—Fe1A—C2A—C3A	−119.9 (4)	C1B—Fe1B—C2B—C3B	−119.6 (5)
C6A—Fe1A—C2A—C3A	46.5 (7)	C7B—Fe1B—C2B—C3B	46.9 (6)
C4A—Fe1A—C2A—C3A	−38.0 (3)	C5B—Fe1B—C2B—C3B	−82.1 (3)
C10A—Fe1A—C2A—C3A	81.4 (3)	C8B—Fe1B—C2B—C3B	−164.9 (6)
C5A—Fe1A—C2A—C1A	38.1 (3)	C10B—Fe1B—C2B—C1B	−118.4 (3)
C7A—Fe1A—C2A—C1A	−40.1 (7)	C4B—Fe1B—C2B—C1B	81.4 (3)
C8A—Fe1A—C2A—C1A	−74.7 (4)	C6B—Fe1B—C2B—C1B	−161.0 (3)
C9A—Fe1A—C2A—C1A	−116.8 (3)	C3B—Fe1B—C2B—C1B	119.6 (5)
C3A—Fe1A—C2A—C1A	119.9 (4)	C9B—Fe1B—C2B—C1B	−76.4 (4)
C6A—Fe1A—C2A—C1A	166.4 (5)	C7B—Fe1B—C2B—C1B	166.5 (5)
C4A—Fe1A—C2A—C1A	81.8 (3)	C5B—Fe1B—C2B—C1B	37.5 (3)
C10A—Fe1A—C2A—C1A	−158.7 (3)	C8B—Fe1B—C2B—C1B	−45.3 (8)
C1A—C2A—C3A—C4A	0.6 (5)	C1B—C2B—C3B—C4B	0.6 (6)
Fe1A—C2A—C3A—C4A	59.8 (3)	Fe1B—C2B—C3B—C4B	59.4 (4)
C1A—C2A—C3A—Fe1A	−59.1 (3)	C1B—C2B—C3B—Fe1B	−58.9 (4)
C5A—Fe1A—C3A—C2A	81.3 (3)	C10B—Fe1B—C3B—C2B	−76.3 (4)
C7A—Fe1A—C3A—C2A	160.9 (5)	C4B—Fe1B—C3B—C2B	119.0 (5)
C8A—Fe1A—C3A—C2A	−39.0 (7)	C6B—Fe1B—C3B—C2B	−118.2 (3)
C9A—Fe1A—C3A—C2A	−75.8 (4)	C9B—Fe1B—C3B—C2B	−43.4 (8)
C1A—Fe1A—C3A—C2A	37.4 (3)	C1B—Fe1B—C3B—C2B	37.4 (3)
C6A—Fe1A—C3A—C2A	−161.7 (3)	C7B—Fe1B—C3B—C2B	−160.0 (3)
C4A—Fe1A—C3A—C2A	118.8 (4)	C5B—Fe1B—C3B—C2B	81.0 (3)
C10A—Fe1A—C3A—C2A	−118.3 (3)	C8B—Fe1B—C3B—C2B	167.7 (5)
C5A—Fe1A—C3A—C4A	−37.5 (3)	C10B—Fe1B—C3B—C4B	164.7 (3)
C7A—Fe1A—C3A—C4A	42.1 (7)	C6B—Fe1B—C3B—C4B	122.8 (3)
C8A—Fe1A—C3A—C4A	−157.8 (5)	C9B—Fe1B—C3B—C4B	−162.4 (6)
C9A—Fe1A—C3A—C4A	165.4 (3)	C1B—Fe1B—C3B—C4B	−81.6 (3)
C2A—Fe1A—C3A—C4A	−118.8 (4)	C7B—Fe1B—C3B—C4B	81.0 (4)
C1A—Fe1A—C3A—C4A	−81.4 (3)	C5B—Fe1B—C3B—C4B	−38.0 (3)
C6A—Fe1A—C3A—C4A	79.5 (3)	C8B—Fe1B—C3B—C4B	48.7 (7)
C10A—Fe1A—C3A—C4A	122.8 (3)	C2B—Fe1B—C3B—C4B	−119.0 (5)
C2A—C3A—C4A—C5A	−1.0 (5)	C2B—C3B—C4B—C5B	0.0 (6)
Fe1A—C3A—C4A—C5A	58.6 (3)	Fe1B—C3B—C4B—C5B	60.2 (3)
C2A—C3A—C4A—Fe1A	−59.6 (3)	C2B—C3B—C4B—Fe1B	−60.2 (4)
C7A—Fe1A—C4A—C5A	77.7 (4)	C10B—Fe1B—C4B—C5B	−160.3 (6)
C8A—Fe1A—C4A—C5A	42.5 (6)	C6B—Fe1B—C4B—C5B	165.5 (3)
C9A—Fe1A—C4A—C5A	−161.6 (6)	C3B—Fe1B—C4B—C5B	−118.5 (5)
C2A—Fe1A—C4A—C5A	−81.5 (3)	C9B—Fe1B—C4B—C5B	46.9 (7)
C1A—Fe1A—C4A—C5A	−37.6 (3)	C1B—Fe1B—C4B—C5B	−37.5 (3)
C3A—Fe1A—C4A—C5A	−119.3 (4)	C7B—Fe1B—C4B—C5B	123.6 (3)
C6A—Fe1A—C4A—C5A	120.7 (3)	C8B—Fe1B—C4B—C5B	81.6 (4)
C10A—Fe1A—C4A—C5A	162.8 (3)	C2B—Fe1B—C4B—C5B	−81.1 (3)
C5A—Fe1A—C4A—C3A	119.3 (4)	C10B—Fe1B—C4B—C3B	−41.9 (8)
C7A—Fe1A—C4A—C3A	−163.0 (3)	C6B—Fe1B—C4B—C3B	−76.1 (4)
C8A—Fe1A—C4A—C3A	161.8 (5)	C9B—Fe1B—C4B—C3B	165.4 (5)
C9A—Fe1A—C4A—C3A	−42.2 (8)	C1B—Fe1B—C4B—C3B	81.0 (3)
C2A—Fe1A—C4A—C3A	37.8 (3)	C7B—Fe1B—C4B—C3B	−118.0 (3)
C1A—Fe1A—C4A—C3A	81.7 (3)	C5B—Fe1B—C4B—C3B	118.5 (5)
C6A—Fe1A—C4A—C3A	−119.9 (3)	C8B—Fe1B—C4B—C3B	−160.0 (3)
C10A—Fe1A—C4A—C3A	−77.8 (4)	C2B—Fe1B—C4B—C3B	37.4 (3)
C3A—C4A—C5A—C1A	0.9 (5)	C2B—C1B—C5B—C4B	0.9 (6)
Fe1A—C4A—C5A—C1A	59.8 (3)	Fe1B—C1B—C5B—C4B	−59.1 (4)
C3A—C4A—C5A—Fe1A	−58.8 (3)	C2B—C1B—C5B—Fe1B	60.0 (4)
C2A—C1A—C5A—C4A	−0.5 (5)	C3B—C4B—C5B—C1B	−0.5 (6)
Fe1A—C1A—C5A—C4A	−60.1 (3)	Fe1B—C4B—C5B—C1B	59.4 (3)
C2A—C1A—C5A—Fe1A	59.6 (3)	C3B—C4B—C5B—Fe1B	−59.9 (4)
C7A—Fe1A—C5A—C4A	−119.8 (3)	C10B—Fe1B—C5B—C1B	44.5 (6)
C8A—Fe1A—C5A—C4A	−161.7 (3)	C4B—Fe1B—C5B—C1B	−119.2 (4)
C9A—Fe1A—C5A—C4A	166.1 (5)	C6B—Fe1B—C5B—C1B	−159.6 (6)
C2A—Fe1A—C5A—C4A	81.5 (3)	C3B—Fe1B—C5B—C1B	−80.9 (3)
C1A—Fe1A—C5A—C4A	119.5 (4)	C9B—Fe1B—C5B—C1B	80.6 (3)
C3A—Fe1A—C5A—C4A	37.8 (3)	C7B—Fe1B—C5B—C1B	164.8 (3)
C6A—Fe1A—C5A—C4A	−78.6 (4)	C8B—Fe1B—C5B—C1B	123.1 (3)
C10A—Fe1A—C5A—C4A	−48.8 (7)	C2B—Fe1B—C5B—C1B	−37.5 (3)
C7A—Fe1A—C5A—C1A	120.6 (3)	C10B—Fe1B—C5B—C4B	163.7 (5)
C8A—Fe1A—C5A—C1A	78.8 (3)	C6B—Fe1B—C5B—C4B	−40.4 (8)
C9A—Fe1A—C5A—C1A	46.6 (6)	C3B—Fe1B—C5B—C4B	38.3 (3)
C2A—Fe1A—C5A—C1A	−38.0 (3)	C9B—Fe1B—C5B—C4B	−160.3 (3)
C3A—Fe1A—C5A—C1A	−81.8 (3)	C1B—Fe1B—C5B—C4B	119.2 (4)
C6A—Fe1A—C5A—C1A	161.9 (3)	C7B—Fe1B—C5B—C4B	−76.1 (4)
C4A—Fe1A—C5A—C1A	−119.5 (4)	C8B—Fe1B—C5B—C4B	−117.7 (3)
C10A—Fe1A—C5A—C1A	−168.3 (6)	C2B—Fe1B—C5B—C4B	81.6 (3)
C5A—Fe1A—C6A—C7A	−75.2 (4)	C10B—Fe1B—C6B—C7B	118.7 (4)
C8A—Fe1A—C6A—C7A	37.7 (3)	C4B—Fe1B—C6B—C7B	−77.7 (3)
C9A—Fe1A—C6A—C7A	81.6 (3)	C3B—Fe1B—C6B—C7B	−119.3 (3)
C2A—Fe1A—C6A—C7A	165.9 (5)	C9B—Fe1B—C6B—C7B	80.5 (3)
C1A—Fe1A—C6A—C7A	−39.9 (7)	C1B—Fe1B—C6B—C7B	167.4 (5)
C3A—Fe1A—C6A—C7A	−159.8 (3)	C5B—Fe1B—C6B—C7B	−47.1 (7)
C4A—Fe1A—C6A—C7A	−117.2 (3)	C8B—Fe1B—C6B—C7B	37.1 (3)
C10A—Fe1A—C6A—C7A	118.8 (4)	C2B—Fe1B—C6B—C7B	−160.8 (3)
C5A—Fe1A—C6A—C10A	166.0 (3)	C4B—Fe1B—C6B—C10B	163.6 (3)
C7A—Fe1A—C6A—C10A	−118.8 (4)	C3B—Fe1B—C6B—C10B	121.9 (3)
C8A—Fe1A—C6A—C10A	−81.1 (3)	C9B—Fe1B—C6B—C10B	−38.2 (3)
C9A—Fe1A—C6A—C10A	−37.2 (3)	C1B—Fe1B—C6B—C10B	48.6 (6)
C2A—Fe1A—C6A—C10A	47.1 (7)	C7B—Fe1B—C6B—C10B	−118.7 (4)
C1A—Fe1A—C6A—C10A	−158.7 (5)	C5B—Fe1B—C6B—C10B	−165.8 (6)
C3A—Fe1A—C6A—C10A	81.4 (3)	C8B—Fe1B—C6B—C10B	−81.6 (3)
C4A—Fe1A—C6A—C10A	123.9 (3)	C2B—Fe1B—C6B—C10B	80.5 (3)
C10A—C6A—C7A—C8A	0.0 (5)	C10B—C6B—C7B—C8B	−0.3 (5)
Fe1A—C6A—C7A—C8A	−59.7 (3)	Fe1B—C6B—C7B—C8B	−59.3 (3)
C10A—C6A—C7A—Fe1A	59.6 (3)	C10B—C6B—C7B—Fe1B	59.1 (3)
C5A—Fe1A—C7A—C8A	−117.8 (3)	C10B—Fe1B—C7B—C8B	81.6 (3)
C9A—Fe1A—C7A—C8A	37.4 (3)	C4B—Fe1B—C7B—C8B	−118.5 (3)
C2A—Fe1A—C7A—C8A	−46.6 (7)	C6B—Fe1B—C7B—C8B	119.9 (4)
C1A—Fe1A—C7A—C8A	−76.4 (3)	C3B—Fe1B—C7B—C8B	−161.4 (3)
C3A—Fe1A—C7A—C8A	170.0 (5)	C9B—Fe1B—C7B—C8B	37.1 (3)
C6A—Fe1A—C7A—C8A	119.1 (4)	C1B—Fe1B—C7B—C8B	−43.9 (8)
C4A—Fe1A—C7A—C8A	−159.1 (3)	C5B—Fe1B—C7B—C8B	−76.9 (3)
C10A—Fe1A—C7A—C8A	81.0 (3)	C2B—Fe1B—C7B—C8B	165.1 (5)
C5A—Fe1A—C7A—C6A	123.1 (3)	C10B—Fe1B—C7B—C6B	−38.2 (3)
C8A—Fe1A—C7A—C6A	−119.1 (4)	C4B—Fe1B—C7B—C6B	121.6 (3)
C9A—Fe1A—C7A—C6A	−81.7 (3)	C3B—Fe1B—C7B—C6B	78.8 (3)
C2A—Fe1A—C7A—C6A	−165.7 (5)	C9B—Fe1B—C7B—C6B	−82.8 (3)
C1A—Fe1A—C7A—C6A	164.6 (3)	C1B—Fe1B—C7B—C6B	−163.8 (6)
C3A—Fe1A—C7A—C6A	50.9 (6)	C5B—Fe1B—C7B—C6B	163.2 (3)
C4A—Fe1A—C7A—C6A	81.8 (3)	C8B—Fe1B—C7B—C6B	−119.9 (4)
C10A—Fe1A—C7A—C6A	−38.1 (3)	C2B—Fe1B—C7B—C6B	45.2 (6)
C6A—C7A—C8A—C9A	0.8 (5)	C6B—C7B—C8B—C9B	0.2 (5)
Fe1A—C7A—C8A—C9A	−59.0 (3)	Fe1B—C7B—C8B—C9B	−58.9 (3)
C6A—C7A—C8A—Fe1A	59.8 (3)	C6B—C7B—C8B—Fe1B	59.0 (3)
C5A—Fe1A—C8A—C7A	79.5 (3)	C10B—Fe1B—C8B—C7B	−81.6 (3)
C9A—Fe1A—C8A—C7A	−119.9 (4)	C4B—Fe1B—C8B—C7B	80.1 (3)
C2A—Fe1A—C8A—C7A	161.9 (3)	C6B—Fe1B—C8B—C7B	−37.4 (3)
C1A—Fe1A—C8A—C7A	121.1 (3)	C3B—Fe1B—C8B—C7B	44.7 (6)
C3A—Fe1A—C8A—C7A	−168.8 (5)	C9B—Fe1B—C8B—C7B	−120.1 (4)
C6A—Fe1A—C8A—C7A	−37.9 (3)	C1B—Fe1B—C8B—C7B	164.6 (3)
C4A—Fe1A—C8A—C7A	49.0 (6)	C5B—Fe1B—C8B—C7B	122.9 (3)
C10A—Fe1A—C8A—C7A	−82.1 (3)	C2B—Fe1B—C8B—C7B	−161.1 (6)
C5A—Fe1A—C8A—C9A	−160.6 (3)	C10B—Fe1B—C8B—C9B	38.5 (3)
C7A—Fe1A—C8A—C9A	119.9 (4)	C4B—Fe1B—C8B—C9B	−159.8 (3)
C2A—Fe1A—C8A—C9A	−78.1 (4)	C6B—Fe1B—C8B—C9B	82.7 (3)
C1A—Fe1A—C8A—C9A	−118.9 (3)	C3B—Fe1B—C8B—C9B	164.8 (5)
C3A—Fe1A—C8A—C9A	−48.8 (7)	C1B—Fe1B—C8B—C9B	−75.3 (4)
C6A—Fe1A—C8A—C9A	82.0 (3)	C7B—Fe1B—C8B—C9B	120.1 (4)
C4A—Fe1A—C8A—C9A	168.9 (5)	C5B—Fe1B—C8B—C9B	−117.0 (3)
C10A—Fe1A—C8A—C9A	37.8 (3)	C2B—Fe1B—C8B—C9B	−41.0 (8)
C7A—C8A—C9A—C10A	−1.3 (5)	C7B—C8B—C9B—C10B	0.0 (5)
Fe1A—C8A—C9A—C10A	−60.2 (3)	Fe1B—C8B—C9B—C10B	−58.9 (3)
C7A—C8A—C9A—Fe1A	58.9 (3)	C7B—C8B—C9B—Fe1B	59.0 (3)
C5A—Fe1A—C9A—C8A	44.9 (6)	C10B—Fe1B—C9B—C8B	−118.7 (4)
C7A—Fe1A—C9A—C8A	−37.3 (3)	C4B—Fe1B—C9B—C8B	48.1 (6)
C2A—Fe1A—C9A—C8A	120.9 (3)	C6B—Fe1B—C9B—C8B	−80.7 (3)
C1A—Fe1A—C9A—C8A	78.3 (4)	C3B—Fe1B—C9B—C8B	−162.0 (6)
C3A—Fe1A—C9A—C8A	162.2 (3)	C1B—Fe1B—C9B—C8B	124.1 (3)
C6A—Fe1A—C9A—C8A	−81.3 (3)	C7B—Fe1B—C9B—C8B	−37.0 (3)
C4A—Fe1A—C9A—C8A	−165.4 (6)	C5B—Fe1B—C9B—C8B	82.0 (3)
C10A—Fe1A—C9A—C8A	−118.9 (4)	C2B—Fe1B—C9B—C8B	165.4 (3)
C5A—Fe1A—C9A—C10A	163.7 (5)	C4B—Fe1B—C9B—C10B	166.7 (5)
C7A—Fe1A—C9A—C10A	81.6 (3)	C6B—Fe1B—C9B—C10B	38.0 (3)
C8A—Fe1A—C9A—C10A	118.9 (4)	C3B—Fe1B—C9B—C10B	−43.4 (7)
C2A—Fe1A—C9A—C10A	−120.2 (3)	C1B—Fe1B—C9B—C10B	−117.3 (3)
C1A—Fe1A—C9A—C10A	−162.8 (3)	C7B—Fe1B—C9B—C10B	81.6 (3)
C3A—Fe1A—C9A—C10A	−78.9 (4)	C5B—Fe1B—C9B—C10B	−159.4 (3)
C6A—Fe1A—C9A—C10A	37.6 (3)	C8B—Fe1B—C9B—C10B	118.7 (4)
C4A—Fe1A—C9A—C10A	−46.6 (8)	C2B—Fe1B—C9B—C10B	−75.9 (3)
C8A—C9A—C10A—C6A	1.3 (5)	C7B—C6B—C10B—C9B	0.3 (5)
Fe1A—C9A—C10A—C6A	−58.4 (3)	Fe1B—C6B—C10B—C9B	60.0 (3)
C8A—C9A—C10A—C11A	−177.9 (4)	C7B—C6B—C10B—C11B	−172.7 (4)
Fe1A—C9A—C10A—C11A	122.4 (5)	Fe1B—C6B—C10B—C11B	−113.0 (5)
C8A—C9A—C10A—Fe1A	59.7 (3)	C7B—C6B—C10B—Fe1B	−59.7 (3)
C7A—C6A—C10A—C9A	−0.8 (5)	C8B—C9B—C10B—C6B	−0.2 (5)
Fe1A—C6A—C10A—C9A	58.3 (3)	Fe1B—C9B—C10B—C6B	−60.0 (3)
C7A—C6A—C10A—C11A	178.4 (4)	C8B—C9B—C10B—C11B	172.8 (5)
Fe1A—C6A—C10A—C11A	−122.5 (5)	Fe1B—C9B—C10B—C11B	113.0 (5)
C7A—C6A—C10A—Fe1A	−59.1 (3)	C8B—C9B—C10B—Fe1B	59.8 (3)
C5A—Fe1A—C10A—C9A	−159.0 (6)	C4B—Fe1B—C10B—C6B	−45.2 (7)
C7A—Fe1A—C10A—C9A	−81.8 (3)	C3B—Fe1B—C10B—C6B	−76.9 (3)
C8A—Fe1A—C10A—C9A	−38.0 (3)	C9B—Fe1B—C10B—C6B	118.9 (4)
C2A—Fe1A—C10A—C9A	78.5 (3)	C1B—Fe1B—C10B—C6B	−159.5 (3)
C1A—Fe1A—C10A—C9A	40.9 (6)	C7B—Fe1B—C10B—C6B	37.8 (3)
C3A—Fe1A—C10A—C9A	121.4 (3)	C5B—Fe1B—C10B—C6B	168.5 (5)
C6A—Fe1A—C10A—C9A	−119.8 (4)	C8B—Fe1B—C10B—C6B	81.2 (3)
C4A—Fe1A—C10A—C9A	164.0 (3)	C2B—Fe1B—C10B—C6B	−117.8 (3)
C5A—Fe1A—C10A—C6A	−39.2 (7)	C4B—Fe1B—C10B—C9B	−164.1 (6)
C7A—Fe1A—C10A—C6A	38.0 (3)	C6B—Fe1B—C10B—C9B	−118.9 (4)
C8A—Fe1A—C10A—C6A	81.9 (3)	C3B—Fe1B—C10B—C9B	164.3 (3)
C9A—Fe1A—C10A—C6A	119.8 (4)	C1B—Fe1B—C10B—C9B	81.6 (3)
C2A—Fe1A—C10A—C6A	−161.6 (3)	C7B—Fe1B—C10B—C9B	−81.1 (3)
C1A—Fe1A—C10A—C6A	160.8 (5)	C5B—Fe1B—C10B—C9B	49.6 (6)
C3A—Fe1A—C10A—C6A	−118.7 (3)	C8B—Fe1B—C10B—C9B	−37.7 (3)
C4A—Fe1A—C10A—C6A	−76.2 (4)	C2B—Fe1B—C10B—C9B	123.4 (3)
C5A—Fe1A—C10A—C11A	78.9 (8)	C4B—Fe1B—C10B—C11B	75.1 (8)
C7A—Fe1A—C10A—C11A	156.0 (5)	C6B—Fe1B—C10B—C11B	120.4 (5)
C8A—Fe1A—C10A—C11A	−160.1 (5)	C3B—Fe1B—C10B—C11B	43.5 (5)
C9A—Fe1A—C10A—C11A	−122.1 (6)	C9B—Fe1B—C10B—C11B	−120.8 (5)
C2A—Fe1A—C10A—C11A	−43.6 (5)	C1B—Fe1B—C10B—C11B	−39.1 (5)
C1A—Fe1A—C10A—C11A	−81.2 (7)	C7B—Fe1B—C10B—C11B	158.2 (4)
C3A—Fe1A—C10A—C11A	−0.7 (5)	C5B—Fe1B—C10B—C11B	−71.1 (7)
C6A—Fe1A—C10A—C11A	118.0 (6)	C8B—Fe1B—C10B—C11B	−158.4 (4)
C4A—Fe1A—C10A—C11A	41.9 (5)	C2B—Fe1B—C10B—C11B	2.6 (4)
N2A—N1A—C11A—C10A	−179.9 (4)	N2B—N1B—C11B—C10B	−171.3 (4)
C9A—C10A—C11A—N1A	0.6 (8)	C6B—C10B—C11B—N1B	−172.7 (5)
C6A—C10A—C11A—N1A	−178.5 (4)	C9B—C10B—C11B—N1B	15.5 (7)
Fe1A—C10A—C11A—N1A	92.2 (5)	Fe1B—C10B—C11B—N1B	101.5 (5)
N1A—N2A—C12A—N3A	−1.0 (7)	N1B—N2B—C12B—N3B	10.6 (7)
N1A—N2A—C12A—S1A	−178.6 (3)	N1B—N2B—C12B—S1B	−168.9 (3)

### Hydrogen-bond geometry (Å, °)

**Table:**

*D*—H···*A*	*D*—H	H···*A*	*D*···*A*	*D*—H···*A*
N2A—H2AB···S1A^i^	0.86	2.66	3.370 (4)	141
N3A—H3AC···S1B^ii^	0.86	2.46	3.295 (4)	165
N2B—H2BB···S1A^iii^	0.86	2.52	3.298 (4)	151
N3B—H3BC···S1B^iv^	0.86	2.47	3.323 (4)	173
C7A—H7AA···Cg1	0.98	2.90	3.668 (6)	136

Symmetry codes: (i) −*x*+1, −*y*, −*z*; (ii) *x*−1, *y*−1, *z*; (iii) *x*, *y*+1, *z*; (iv) −*x*+2, −*y*+1, −*z*.

## References

[bb1] Bruker (2009). *APEX2*, *SAINT* and *SADABS* Bruker AXS Inc., Madison, Wisconsin, USA.

[bb2] Cosier, J. & Glazer, A. M. (1986). *J. Appl. Cryst.* **19**, 105–107.

[bb3] Mariño, M., Gayoso, E., Antelo, J. M., Adrio, L. A., Fernańdez, J. J. & Vila, J. M. (2006). *Polyhedron*, **25**, 1449–1456.

[bb4] Sheldrick, G. M. (2008). *Acta Cryst.* A**64**, 112–122. 10.1107/S0108767307043930 18156677

[bb5] Spek, A. L. (2009). *Acta Cryst.* D**65**, 148–155. 10.1107/S090744490804362X PMC2631630 19171970

[bb6] Vikneswaran, M. R., Teoh, S. G., Yeap, C. S. & Fun, H.-K. (2009). *Acta Cryst.* E**65**, m1524–m1525. 10.1107/S1600536809046078 PMC2971859 21578570

